# Empowering semiconductor chips via diamond-integrated near-junction cooling

**DOI:** 10.1093/nsr/nwag482

**Published:** 2026-08-12

**Authors:** Wenjun Liang, Yang Lu

**Affiliations:** Department of Mechanical Engineering, The University of Hong Kong, China; Department of Mechanical Engineering, The University of Hong Kong, China

At the historic convergence of the post-Moore era and the exponential AI boom, the reliance of high-performance computing on 3D heterogeneous architectures has elevated nanoscale interfacial bonding and thermal transport into a paramount research frontier [1]. Diamond has the remarkable ability to keep chips cool due to its highest thermal conductivity. Furthermore, diamond holds immense potential for crosstalk and leakage prevention in future heterogeneous integration owing to its ultrawide bandgap and ultrahigh breakdown field strength. These properties make diamond a premier candidate for next-generation semiconductor technology. Meanwhile, the rapid expansion of power electronics drives an urgent demand for beta-phase gallium oxide (*β*-Ga_2_O_3_), whose performance is severely constrained by its low intrinsic thermal conductivity. To relieve this thermal localization bottleneck, near-junction integration with diamond offers a highly attractive solution. However, the integration of diamond with *β*-Ga_2_O_3_ faces challenges due to their large lattice mismatch.

To overcome the significant integration challenges, Prof. Chongxin Shan and Prof. Shaobo Cheng’s group at Zhengzhou University, in collaboration with Prof. Peng Gao at Peking University, established an innovative gallium-mediated epitaxial pathway via chemical vapor deposition [2]. The method is easily accessible and remarkably effective. Heterointerface is atomically sharp and covalently bonding and shows remarkably low thermal boundary resistance and high bonding strength. These factors are significant for heat transfer performance and chip stability, verifying this technique as a pivotal reference for future diamond-based heterogeneous integration.

Beyond the novel integration strategy, this study provides atomic-level physical insights into the mechanics of phonon-driven heat transport at heterointerface. The coupling of high-resolution transmission electron microscopy (HRTEM) and vibrational electron energy loss spectroscopy (EELS) advances the paradigm of interfacial thermal transport research, shifting it from speculative theoretical simulations to definitive verification in real space, achieving a monumental breakthrough in atomic-resolution observation.

**Figure 1.**
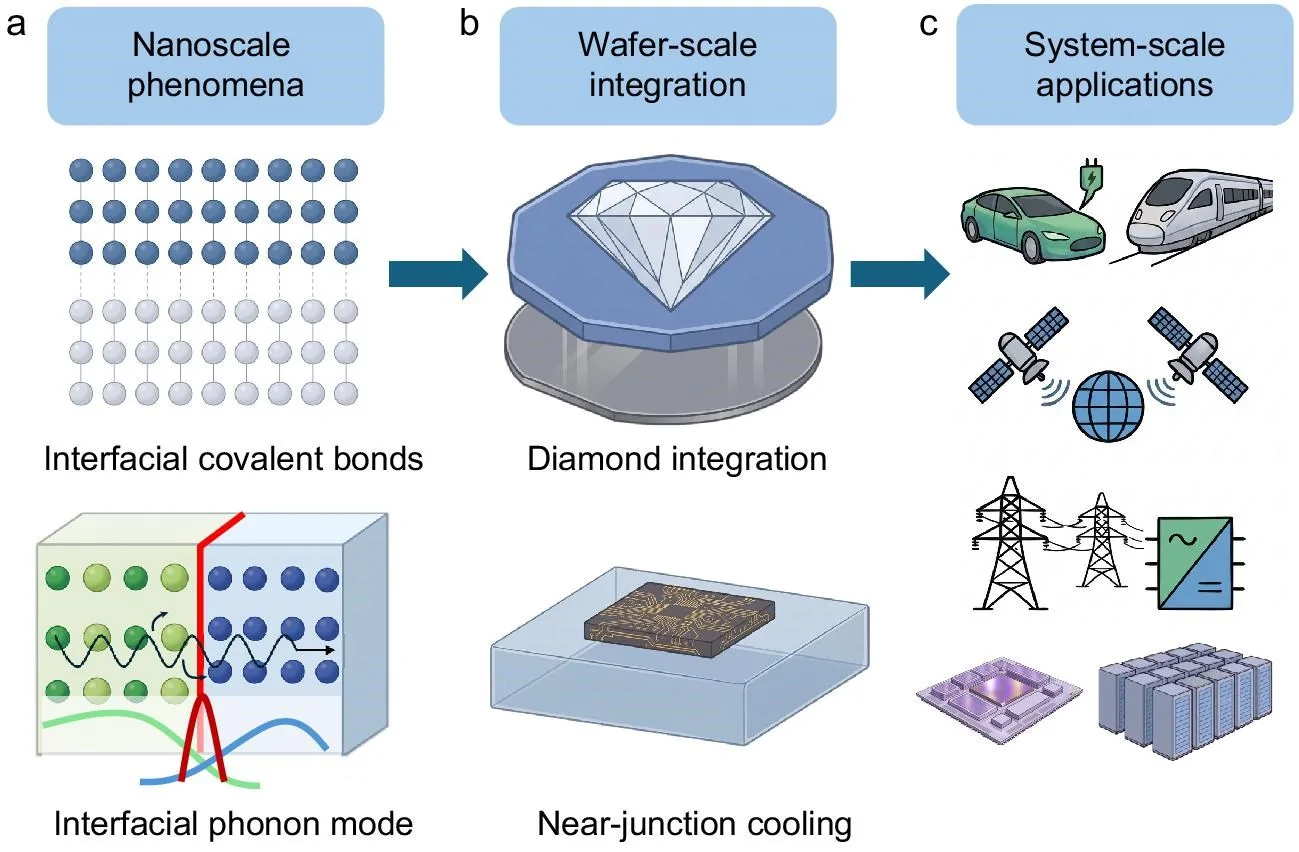
Multiscale framework of diamond-integrated thermal management solution. (a) Schematic representations of nanoscale interfacial covalent bonding and the associated phonon mode mismatch and coupling mechanisms. (b) Wafer-scale diamond integration techniques, enabling near-junction thermal management and local hot-spot dissipation. (c) Representative applications in electric transportation, satellite communications, power grid systems, integrated circuits, and AI data centers.

This work will further inspire chip-level thermal management solutions via interfacial covalent bonding and phonon mode transport, as shown in Fig. 1a. Building upon these nanoscale physical insights, wafer-scale diamond integration is achieved to provide efficient near-junction cooling for other semiconductor materials (Fig. 1b). By providing unparalleled heat dissipation, diamond drives unprecedented performance breakthroughs in existing materials and vastly extends their operational limits. Similarly, for emerging materials (GaN, *β*-Ga_2_O_3_, AlN, AlGaN, ZnO, and 2D materials), diamond integration overcomes severe thermal bottlenecks, unlocking their potential in high-voltage power electronics, radio-frequency devices, and next-generation logic chips (Fig. 1c). Notably, microfabricated single-crystal diamond has been demonstrated to sustain ultra-large elastic strain, offering profound modulation of its electronic band structure [3]. Looking ahead, combining such heterogeneous integration with deep elastic strain engineering of diamond could further offer an exciting avenue for actively driving phonon transport and dynamically tuning interfacial heat dissipation [4,5].

In summary, this work provides a new strategy for constructing heterogeneous interfaces that unite high interfacial bond strength with exceptional thermal conductance, thereby paving the way for diamond-integrated thermal management solutions across next-generation power electronics, high-frequency devices, and advanced quantum platforms.
